# Angioedema From Triple Therapy: A Case Report

**DOI:** 10.7759/cureus.46247

**Published:** 2023-09-30

**Authors:** Nicholas Lepore, Taya Carpenter, Alan Wolff

**Affiliations:** 1 Internal Medicine - Pediatrics, Rutgers New Jersey Medical School, Newark, USA; 2 Pulmonary & Critical Care Medicine and Allergy & Rheumatology, Rutgers New Jersey Medical School, Newark, USA

**Keywords:** calcium channel blocker, radiocontrast, amlodipine, alogliptin, lisinopril, dpp-iv inhibitor, ace inhibitor, angioedema

## Abstract

Angioedema is a rare but potentially life-threatening complication associated with angiotensin-converting enzyme (ACE) inhibitors. Although the pathophysiology is well understood, cases involving the concurrent use of other medications are less explored. We present a unique case of ACE inhibitor-induced angioedema in a 57-year-old male, which developed soon after receiving intravenous contrast. The patient's medication list included a dipeptidyl peptidase-IV inhibitor and a calcium channel blocker. Studies have shown an increased risk of angioedema with the combined use of these medications, likely due to alterations in bradykinin metabolism. This case highlights the importance of medication review and consideration of potential drug interactions when prescribing ACE inhibitors. It emphasizes the significance of diagnostic accuracy to avoid the mislabeling of allergies and consideration of other etiologies in angioedema. Healthcare providers ought to be mindful of the increased risk of angioedema when prescribing dipeptidyl peptidase-IV inhibitors and calcium-channel blockers with ACE inhibitors, as these are frequently used medications.

## Introduction

Angioedema is a well-documented and potentially life-threatening adverse reaction to angiotensin-converting enzyme (ACE) inhibitors [1]. Angioedema has also been reported with several other classes of medications, including dipeptidyl peptidase-IV (DPP-IV) inhibitors, dihydropyridine calcium-channel blockers (CCBs), and nonsteroidal anti-inflammatory drugs (NSAIDs), as individual agents and with increased frequency in combination with ACE inhibitors. Radiocontrast media is another known cause of angioedema, though the histaminergic mechanism differs from the bradykinin-mediated angioedema of the medications previously mentioned [2]. In this case report, we describe a patient who developed angioedema soon after receiving an NSAID and both oral (PO) and intravenous (IV) radiocontrast. The patient had been taking an ACE inhibitor, DPP-IV inhibitor, and dihydropyridine calcium channel blocker for his comorbid conditions.

## Case presentation

A 57-year-old African American male with a history of type II diabetes, essential hypertension, and gastroesophageal reflux disease was admitted for evaluation of abdominal pain, fever, and hyperbilirubinemia concerning for ascending cholangitis. His home medications include lisinopril 40 mg daily, spironolactone 25 mg daily, metformin 500 mg three times daily, alogliptin 12.5 mg daily, amlodipine 10 mg daily, hydrochlorothiazide 12.5 mg daily, and atorvastatin 40 mg daily. He reported compliance with all his medications. He reports smoking a quarter pack of cigarettes daily. He had no known allergies and denied a personal or family history of angioedema. 

**Figure 1:**
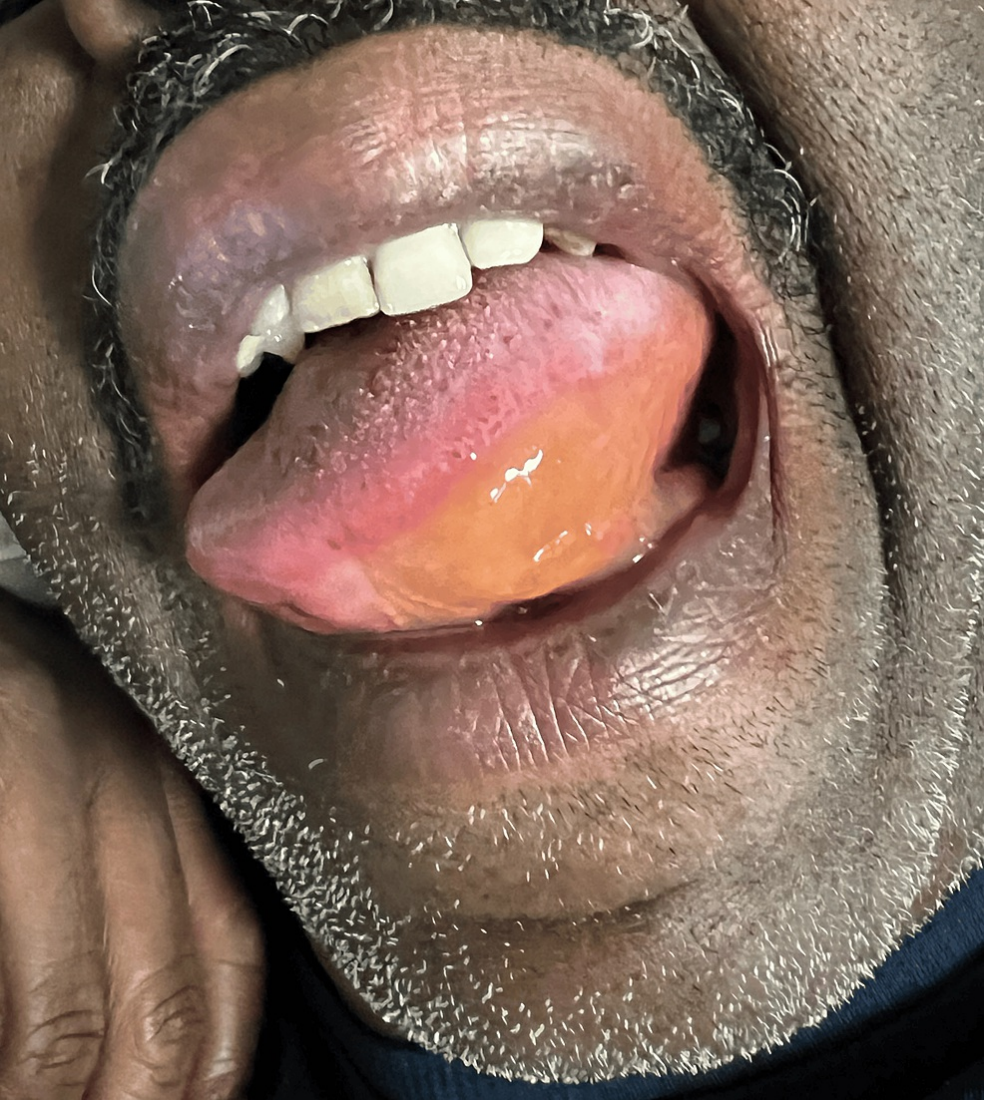
Unilateral tongue swelling

Approximately 30 minutes after undergoing a computed tomography (CT) scan with IV and positive oral (PO) contrast, the patient developed left-sided tongue swelling. He denied lightheadedness, shortness of breath, pruritus, tingling, urticaria, difficulty swallowing secretions, abdominal pain, vomiting, and diarrhea. On physical exam, his vitals were stable, and he appeared comfortable lying in bed. He had significant left-sided tongue edema (Figure 1) and demonstrated an ability to swallow, though with some difficulty. Cardiac and respiratory exams were unremarkable and without stridor or wheezing. An abdominal exam was notable for mild, right-sided upper and lower quadrant tenderness. Extremities showed equal pulses bilaterally without edema. Skin exam was negative for urticaria or other rashes. ENT was consulted but were not available for nasopharyngeal laryngoscopy. The patient was treated with diphenhydramine 50 mg IV, methylprednisolone 125 mg IV, and epinephrine 0.3 mg IM (Epi-Pen) with minimal improvement in his tongue edema. Laboratory workups, including C4 and C3, were normal. Approximately 12 hours later, the patient reported significant improvement in his tongue swelling and did not develop recurrent symptoms. The allergy and immunology team was consulted for further evaluation.

Per medication review, the patient was given oral gastrografin and ibuprofen 400 mg four and two hours, respectively, prior to symptom onset. He received IV Visipaque-320 (iodixanol) approximately thirty minutes before the development of tongue swelling. Per chart review and discussion with the patient, he previously received and tolerated both IV Visipaque-320 and PO Omnipaque-240 on at least three occasions, most recently within the past year.

The patient has been on lisinopril for the past two years, with an increase to the medication's maximum dose about a year prior to his current presentation. He has been on amlodipine since 2007 and was started on alogliptin in 2022, about eight months prior to this episode. The patient's presentation of angioedema with a lack of histaminergic symptoms was concerning for ACE inhibitor-induced angioedema, and the medication was discontinued. He was subsequently started on losartan 50 mg per the primary team.

## Discussion

ACE inhibitor-induced angioedema is a rare complication of therapy with an estimated incidence between 0.1% and 0.7% of treated individuals [1]. ACE inhibitors prevent the metabolic breakdown of bradykinin, an oligopeptide that induces vasodilation, leading to increased vascular permeability [1]. Risk factors for ACE inhibitor-induced angioedema include African descent, age older than 65 years, smoking, and NSAID use [1].

DPP-IV inhibitors, also called gliptins, are a class of oral antidiabetic medications that function by blocking the action of DPP-IV, an enzyme responsible for the degradation of incretins, as well as bradykinin and substance P [3,4]. Postmarketing surveillance of adverse reactions with DPP-IV inhibitors has reported rare occurrences of angioedema [5]. The risk is increased with concurrent use of ACE inhibitors, with a reported odds ratio of 42.77 (95% CI 36.93-49.53) in a disproportionality analysis using the World Health Organization's pharmacovigilance database [6]. Interestingly, the sera of patients with a history of ACE inhibitor-induced angioedema demonstrated a decrease in endogenous DPP-IV enzyme activity compared to patients on ACE inhibitors without angioedema [7]. Measuring DPP-IV enzyme activity may, therefore, assist in identifying patients at increased risk of angioedema, especially prior to prescribing gliptins and ACE inhibitors concurrently.

There are a few case reports in the literature of angioedema attributed to dihydropyridine calcium-channel blockers, such as amlodipine [8]. The mechanism has not been established, though proposed mechanisms involve endothelial release of bradykinin and nitric oxide augmenting arterial vasodilation [9]. Similar to gliptins, when CCBs were concurrently used with ACE inhibitors, there was an increased risk of angioedema, with one large study reporting a 57% increase in risk [10].

## Conclusions

There are several important teaching points to be learned from this case. It is most probable the patient experienced ACE inhibitor-induced angioedema in the setting of multiple risk-enhancing factors, including African ethnicity, active smoking, recent NSAIDs, and concurrent DPP-IV inhibitor and CCB therapy. These medications are routinely used in clinical practice, and healthcare providers ought to be mindful of the increased risks when prescribing DPP-IV inhibitors and CCBs with ACE inhibitors. Another important lesson is to avoid the convenience of inappropriately labeling an allergy based solely on the temporal relationship of exposure to symptom onset. In many instances, it is likely this patient would have been incorrectly diagnosed with a contrast allergy. The patient did not display histaminergic symptoms, and his minimal response to steroids and epinephrine suggests an etiology other than a mast cell-mediated process. This case highlights the importance of medication review and diagnostic accuracy.
